# Glycerol Is an Osmoprotectant in Two Antarctic *Chlamydomonas* Species From an Ice-Covered Saline Lake and Is Synthesized by an Unusual Bidomain Enzyme

**DOI:** 10.3389/fpls.2020.01259

**Published:** 2020-08-20

**Authors:** James A. Raymond, Rachael Morgan-Kiss, Sarah Stahl-Rommel

**Affiliations:** ^1^School of Life Sciences, University of Nevada Las Vegas, Las Vegas, NV, United States; ^2^Department of Microbiology, Miami University, Oxford, OH, United States

**Keywords:** *Chlamydomonas*, glycerol synthesis, phosphoserine phosphatase, glycerol-3-phosphate dehydrogenase, Antarctica, Lake Bonney

## Abstract

Glycerol, a compatible solute, has previously been found to act as an osmoprotectant in some marine *Chlamydomonas* species and several species of *Dunaliella* from hypersaline ponds. Recently, *Chlamydomonas reinhardtii* and *Dunaliella salina* were shown to make glycerol with an unusual bidomain enzyme, which appears to be unique to algae, that contains a phosphoserine phosphatase and glycerol-3-phosphate dehydrogenase. Here we report that two psychrophilic species of *Chlamydomonas* (*C*. spp. UWO241 and ICE-MDV) from Lake Bonney, Antarctica also produce high levels of glycerol to survive in the lake’s saline waters. Glycerol concentration increased linearly with salinity and at 1.3 M NaCl, exceeded 400 mM in *C*. sp. UWO241, the more salt-tolerant strain. We also show that both species expressed several isoforms of the bidomain enzyme. An analysis of one of the isoforms of *C*. sp. UWO241 showed that it was strongly upregulated by NaCl and is thus the likely source of glycerol. These results reveal another adaptation of the Lake Bonney *Chlamydomonas* species that allow them to survive in an extreme polar environment.

## Introduction

Glycerol is well-known for its ability to mitigate environmental stresses such as freezing and hyperosmotic conditions in a wide variety of organisms, including yeast (Wang et al., 2001), insects (Bentz and Mullins, 1999; Bennett et al., 2005), and vertebrates (Raymond, 1992; Layne, 1999). It is also well-known as an osmoprotectant in some algae, including salt pond isolates of *Dunaliella* (Ben-Amotz and Avron, 1973; Sussman and Avron, 1981) and several marine isolates of *Chlamydomonas* (Ahmad and Hellebust, 1986; Miyasaka and Ikeda, 1997; Miyasaka et al., 1998). As a compatible solute, glycerol prevents osmotic water loss and thus a build-up of intracellular salt concentrations. Two psychrophilic *Chlamydomonas* species, *Chlamydomonas* sp. UWO241 (Neale and Priscu, 1995) (hereafter Chlamy-UWO) and *Chlamydomonas* sp. ICE-MDV (Li et al., 2016) (hereafter Chlamy-ICE) were isolated from Lake Bonney, a permanently ice-covered lake in Antarctica. The lake has a steep halocline, in which the salinity reaches about 150 PSU (equivalent to about 2.1 M NaCl, or 4.3 x the salinity of seawater) at a depth of 40 m (Spigel et al., 2018). Chlamy-UWO’s ability to survive in this environment has been intensively studied for over two decades. Among its adaptation are a photochemical apparatus well-suited for a cold, saline and low light environment (Morgan et al., 1998), more fluid membranes (Morgan-Kiss et al., 2002) and ice-binding proteins (IBPs) to prevent freeze-thaw injury (Raymond and Morgan-Kiss, 2013). Less is known about Chlamy-ICE which was isolated more recently: it also possesses IBPs (Raymond and Morgan-Kiss, 2017) but appears to have differences in acclimatory ability compared to Chlamy-UWO (Cook et al., 2019). Much more is known about the cold adaptations of a closely-related Antarctic pyschrophile *Chlamydomonas* sp. ICE-L (Cvetkovska et al., 2017). Because both Chlamy-UWO and Chlamy-ICE are adapted to a saline environment, we wished to know whether they also produce glycerol as an osmoprotectant or cryoprotectant.

Until recently, the pathways used by algae to produce glycerol have been a matter of speculation. Glycogen, or one of its products, dihydroxyacetone phosphate (DHAP), has been identified as the source of glycerol in a number of organisms, including yeast, (Modig et al., 2007), *Arabidopsis* (Caparros-Martin et al., 2007), a bacterium (Larrouy-Maumus et al., 2013), mountain pine beetle (Fraser et al., 2017), and rainbow smelt (Raymond, 1995). In the DHAP pathway, DHAP is converted to glycerol-3-phosphatate (G3P) by a nicotinamide-adenine dinucleotide (NAD^+^)-dependent glycerol-3 phosphate dehydrogenase (GPDH), which is then converted to glycerol by a phosphoserine phosphatase (PSP). The problem is that algae typically have several GPDHs and phosphatases, and attempts to identify the enzymes involved have been until recently unsuccessful.

*Dunaliella salina* has a bidomain enzyme that includes an N-terminal PSP and a C-terminal NAD^+^-dependent GPDH (He et al., 2007). It was proposed as a candidate for glycerol production directly from DHAP (He et al., 2007), but initial attempts to confirm its activity were unsuccessful (He et al., 2009). However, this enzyme still seemed like the most likely source of glycerol. During our study of IBPs in Chlamy-UWO (Raymond and Morgan-Kiss, 2013), we noticed that it produced glycerol as an osmoprotectant, i.e., glycerol production increased with increasing salinity. We searched the Chlamy-UWO genome for a *Dunaliella*-like bidomain enzyme and found at least two homologs. We then examined the expression of one of the homologs and found that it strongly increased with increasing salinity, as reported in a master’s thesis (Stahl, 2014). This suggested that this enzyme alone was capable of producing glycerol from DHAP

Recently, Morales-Sanchez et al. (2017) showed that a similar bidomain enzyme in *Chlamydomonas reinhardtii* was sufficient to produce glycerol directly from DHAP, and He Q. H. et al. (2020) finally confirmed that the *Dunaliella salina* bidomain protein was sufficient to produce glycerol. Here, we report our test of the hypothesis that Chlamy-UWO uses a similar bidomain enzyme to produce glycerol. We also show that Chlamy-ICE is capable of producing high levels of glycerol under saline conditions and has similar bidomain enzymes.

## Methods

### Cells

*Chlamydomonas* sp. UWO241 [Chlamy-UWO, also referred to as *C. raudensis* in some earlier studies, e.g., (Dolhi et al., 2013)] and *Chlamydomonas* sp. ICE-MDV (Chlamy-ICE) were previously isolated from the east lobe of Lake Bonney, Antarctica. Chlamy-UWO was isolated from below the permanent chemocline in the deep photic zone (17 m) where the salinity is similar to the salinity of seawater but rapidly increases with increasing depth (Spigel et al., 2018). Its natural depth range is not known. Chlamy-ICE was recovered from a depth of 13 m, where the salinity is markedly lower (Li et al., 2016). Both strains were maintained in Bold’s basal medium (BBM) supplemented with the indicated amounts of NaCl in a temperature/light regime of 8°C/50 μmol · m^−2^ · s^−1^ at Miami University. *Chlamydomonas* sp. UWO241 is deposited in the Bigelow Algal collection (CCMP1619). *Chlamydomonas* sp. ICE-MDV is available upon request to RMK.

### Glycerol Measurement

Fresh cells were shipped overnight to the University of Nevada Las Vegas (UNLV) with ice packs. The temperature varied by less than 2°C during shipment. For each sample, 1.50 ml of cell culture was centrifuged in pre-weighed tubes. The supernatant was removed and the remaining medium was removed with a fine, drawn-out pipet. The tubes were reweighed to obtain the pellet weights (about 3 mg), sealed, and stored at -25°C. For glycerol measurement, the tubes were thawed, and the bottoms of the tubes were subjected to two freeze-thaw cycles in liquid nitrogen to break the cells. The pellets were suspended in 1.00 ml DI water, vortexed to release glycerol and centrifuged at 16,000 x g for 2 min at 4°C to yield a clear supernatant. Subsequent testing of the pellet showed that virtually all of the glycerol was released from the cells. Glycerol was quantified enzymatically with Free glycerol reagent (Sigma no. F6428), which develops a 540 nm (purple) color in the presence of glycerol. Intracellular glycerol concentration (mM) in a cell pellet was calculated as M_g_ x 1,000/(MW x M_p_ x f), where M_g_ is the mass of glycerol released from the pellet, MW is the molecular weight of glycerol (92 g mol^-1^), M_p_ is the mass of the pellet, and f is fraction of the cell mass that is water. The latter was estimated as 0.7 based on measurements of *Chlamydomonas pulsatilla* (Ahmad and Hellebust, 1986). This calculation assumed that the extracellular water content in the pellets was zero. If extracelular water were present in the pellets, the actual glycerol concentrations would be higher than those reported.

### Gene Sequences

Sequences of the enzymes described in this study were assembled from transcriptome and genome data obtained from Chlamy-UWO (Raymond and Morgan-Kiss, 2013) and Chlamy-ICE (Raymond and Morgan-Kiss, 2017). The transcriptomes were obtained with 454 and Illumina sequencing, respectively. Additional DNA and mRNA reads needed to complete assembly of Chlamy-UWO isoform 3 were kindly provided by David Smith (University of Western Ontario). Gene expression levels were expressed as FPKM (fragments per kilobase per million reads) values: FPKM = r/R/L, where r is the number of unique reads for a given isoform with e-values less than 1e^-20^ (each read was assigned only to the isoform that it most closely matched), R is the number of millions of reads in the transcriptome, and L is the length of the gene in kb. The transcriptomes were searched for genes similar to the bidomain enzyme in *Dunaliella salina* (AAX56341). Several isoforms of the gene were found and assembled. Introns were identified by comparing these sequences with the genomic data. Sequences for the qPCR reference genes were obtained from the Chlamy-UWO transcriptome.

Chloroplast signals were predicted with ChloroP 1.1 (http://www.cbs.dtu.dk/services/ChloroP/) (Emanuelsson et al., 1999). PSP and GPDH domains were identified with NCBI’s conserved domain database https://www.ncbi.nlm.nih.gov/Structure/cdd/wrpsb.cgi. A Neighbor-Joining phylogenetic tree was constructed with Mega X (Tamura et al., 2011), using two non-chlorophyte bidomain proteins to root the tree. We thank Armin Hallmann (University of Bielefeld, Germany) for correcting the bidomain sequence of *Volvox carteri* used in the tree.

### Quantitative PCR

Cultures of Chlamy-UWO were grown at 8°C and 50 μmol m^-2^ s^-1^ in BBM supplemented with a range of salt concentrations (10, 300, 700, and 1,300 mM NaCl) until samples reached the mid exponential phase. Total RNA was extracted with a Qiagen RNeasy mini kit (No. 74104) as per manufacturer’s instructions. Residual genomic DNA was removed by Ambion DNase (Thermo Fisher Scientific, Waltham, MA). RNA was reverse transcribed with an iScript cDNA synthesis kit (Bio-Rad, Hercules, CA) as specified by the manufacturer. Expression of the Chlamy-UWO PSP-GPDH isoform 2 was quantified by real-time quantitative PCR using the ΔΔCq method (Liu et al., 2012) and a Bio-Rad CFX Connect Real-time thermal cycler. Histone H2B (histh2b) and 40S ribosomal protein S10 (rps10) (GenBank accessions MT362546 and MT362547, respectively) were used as reference genes, as their expressions were fairly stable over the conditions used. Primers are shown in **Table S1**.

### Structure Prediction

A 3D model of Chlamy-UWO isoform 1 was predicted with Swiss Model (Waterhouse et al., 2018) (https://swissmodel.expasy.org/) using the structure of the *Dunaliella salina* bidomain protein (6iuy.1.A) (He Q. H. et al., 2020) as template. In the 580-a.a. region of overlap, the two proteins had an identity of 54%. The free energy of the model was then minimized (from -22.8 to -30.9 MJ/mol) with the Yasara energy minimization server (Krieger et al., 2009) (http://www.yasara.org/minimizationserver.htm) and displayed with the Yasara viewer (Waterhouse et al., 2018) (http://www.yasara.org/). Stereo views were obtained by rotating the molecules 3° around the vertical axis.

## Results

**Figure 1:**
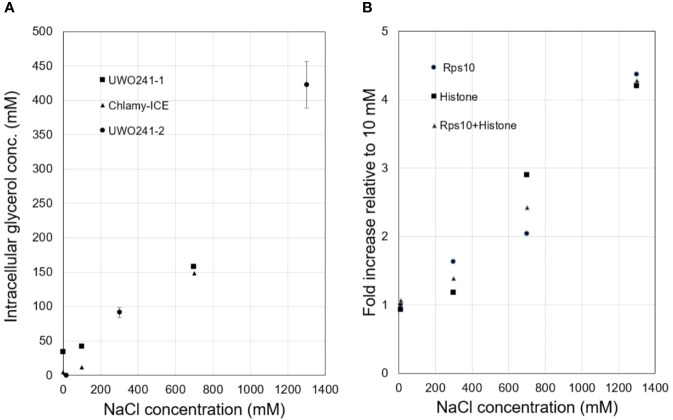
Responses of Lake Bonney *Chlamydomonas* species to increasing salinity. **(A)**, Glycerol production in Chlamy-UWO and Chlamy-ICE. Two independent measurements were made for Chlamy-UWO. For UWO-2, three measurements were made for each sample. **(B)**, Expression of PSP/GPDH isoform 2 of Chlamy-UWO using different reference genes.

Intracellular glycerol levels in Chlamy-UWO and Chlamy-ICE increased with increasing NaCl concentration. Both species maintained glycerol levels of about 150 mM at 700 mM NaCl (**Figure 1A**). At 1,300 mM NaCl, Chlamy-UWO cells reached over 400 mM glycerol while Chlamy-ICE cells were unable to grow. In contrast, glycerol levels in the supernatant of centrifuged dense cultures of Chlamy-UWO were very low (0.4 mM at 700 m NaCl and 1.1 mM at 1,300 mM NaCl), indicating that the cells were maintaining a strong gradient between the intracellular and extracellular environments.

We thus searched the Chlamy-UWO transcriptome for enzymes similar to the proposed PSP/GPDH that had been found in *Dunaliella* (He et al., 2007). Three complete isoforms could be assembled. We selected one of the isoforms to see if it could account for the increased glycerol production at increased salinity. The mRNA expression of Chlamy-UWO isoform 2 increased about four-fold as the NaCl concentration increased to 1,300 mM, regardless of the reference gene (**Figure 1B**). For the reference gene combination Rps10+Histone, whose points were more linear than those of the single reference genes, the increase was significant at the p <0.01 level (linear regression). This increase closely paralleled the increase in glycerol concentration. This, together with the recent findings in *C. reinhardtii* and *D. salina*, strongly supports the idea that the bidomain enzymes are a major source (and possibly the main source) of glycerol in Chlamy-UWO.

When the Chlamy-ICE transcriptome became available in 2016, it was also examined for homologs of the bidomain proteins. It appeared to have seven such homologs, but only five of them could be completely assembled (**Table 1**). For each of the Lake Bonney species, the expression levels of the different isoforms varied considerably (**Table 1**). In the approximately 600-a.a. conserved region that includes both enzyme domains, the identities of the Chlamy-UWO and Chlamy-ICE isoforms to *C. reinhardtii* GPD3 are about 60% (**Table 1**). To gain insights into the relationships of the enzymes in the three *Chlamydomonas* species, their exon/intron structures were compared. The numbers of introns (and their locations) differed considerably among the three *Chlamydomonas* species (**Table 1**), suggesting that their divergence occurred long ago.

**Table 1:** Bidomain PSP/GPDH proteins in three species of *Chlamydomonas*.

Species/isoform	GenBank acc. no.	Relative Expression level^1^	Length (a.a.)	No. exons	% ID (% similarity)^2^
Chlamy-UWO-1	MT362548	13.7	745	18	61 (75)
Chlamy-UWO-2	MT362549	38.4	710	18	62 (77)
Chlamy-UWO-3	MT362550	1.0	741	18	60 (76)
Chlamy-ICE-1	MT362551	1.0	697	11	59 (77)
Chlamy-ICE-2	MT362552	1.7	693	13	56 (75)
Chlamy-ICE-3	MT362553	8.2	665	12	58 (74)
Chlamy-ICE-4	MT362554	6.3	710	7	58 (76)
Chlamy-ICE-5	MT362555	9.0	689	3	62 (77)
*C. reinhardtii* GPD3	AJG44150	N/A	725	15	100 (100)
*C. reinhardtii* GPD2	AJG44149	N/A	723	15	99.8 (99.1)

^1^Reads (transcripts) per kilobase per million mapped reads relative to expression of the most weakly expressed isoform in each species. Culture conditions were 8°C and 700 mM NaCl (Chlamy-UWO) and 500 mM NaCl (Chlamy-ICE). N/A, data not available.

^2^Percent identity (% similarity) to PSP+GPDH region of C. reinhardtii GPD3.

**Figure 2:**
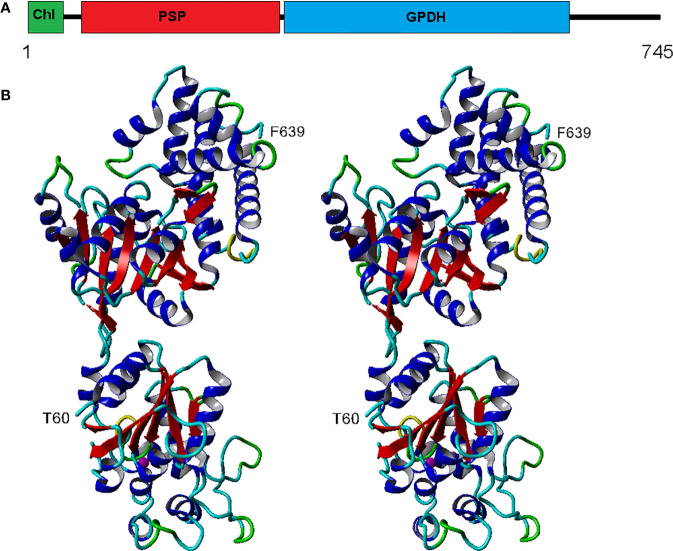
Structure of Chlamy-UWO PSP/GPDH isoform 1. **(A)**, Domain structure, consisting of an N-terminal chloroplast-targeting signal, a phosphoserine phosphatase domain and a glycerol-3-phosphate dehydrogenase domain. **(B)**, Stereoview of the molecule generated by Swiss-Model using the PSP/GPDH of *D. salina* as template. The lower portion, beginning at Thr60, shows the PSP domain with an embedded magnesium ion (olive). The upper portion, ending at Phe639, shows the GPDH domain. Color codes: red, beta sheet; blue, alpha helix; cyan, coil; green, turn; yellow, 3_10_ helix.

The eight complete isoforms of Chlamy-UWO and Chlamy-ICE consist of an N-terminal chloroplast-targeting signal, a PSP domain and a C-terminal NAD^+^-dependent GDPH domain (**Figure 2A**), similar to the structure in *Dunaliella* (He Q. H. et al., 2020). A 3D model of isoform 1 of the Chlamy-UWO enzyme predicted from the structure of the *D. salina* enzyme is shown in **Figure 2B**. The region that could be modeled ranged from Thr60 at the start of the PSP domain (lower part of molecule in **Figure 2B**) to Phe639 near the end of the GPDH domain (upper part). The model is similar to the structure of the *Dunaliella* protein, with two nearly independent domains connected by a short link. The key residues forming the binding sites of DHAP, NAD^+^, and glycerol-3-P in *Dunaliella* (He Q. H. et al., 2020) are conserved in the Chlamy-UWO isoform (**Figure S1**). As a test of the accuracy of the model, the predicted binding sites of DHAP and NAD^+^ on the GPDH domain of the *Chlamydomonas* protein were compared to those in *Dunaliella*. The residues that form the binding sites in the two structures as well as their locations are nearly the same (**Figure S2**), supporting the accuracy of the model.

**Figure 3:**
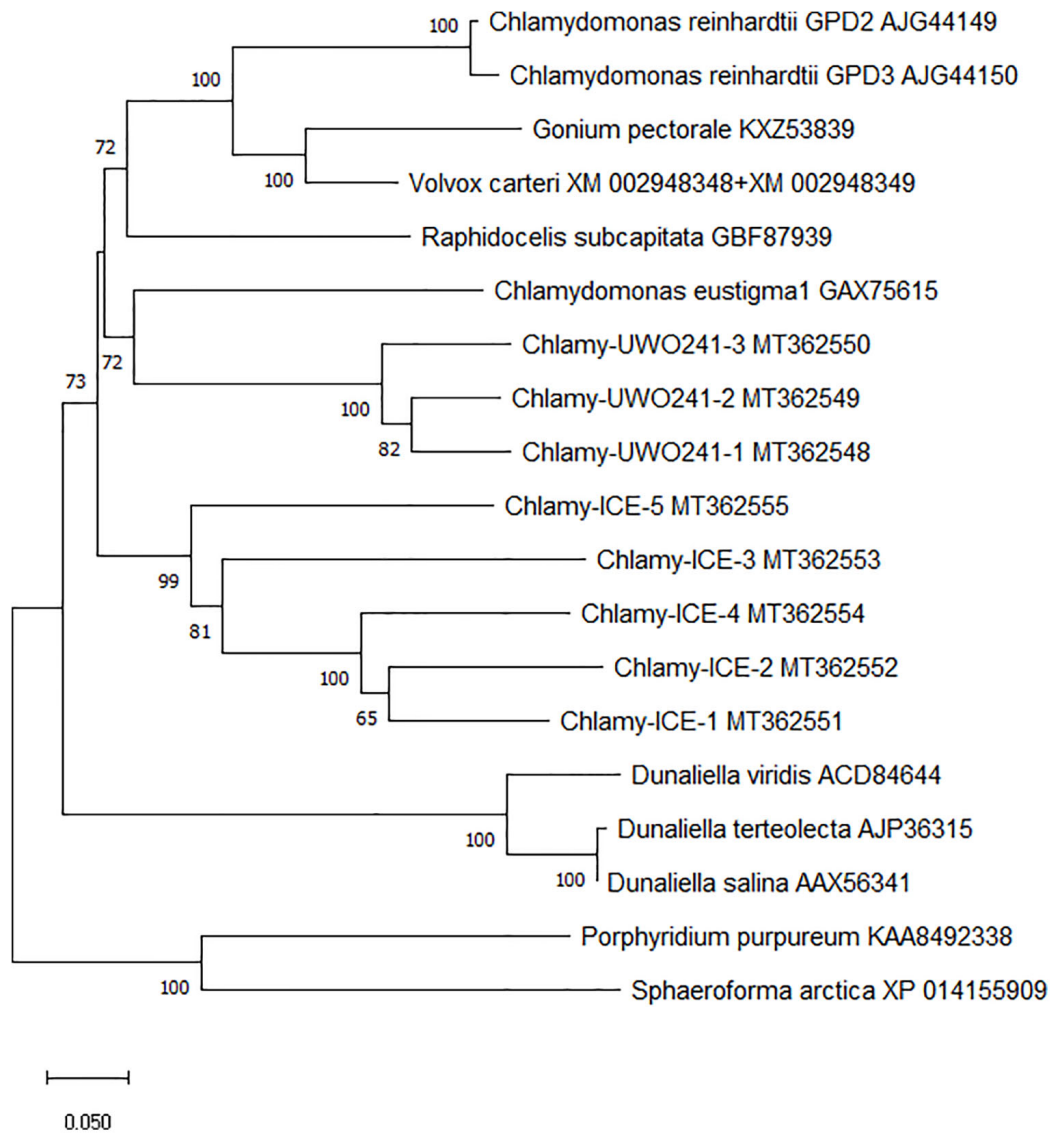
Neighbor-joining phylogenetic tree of the ~600 a.a. region containing the PSP and GPDH domains of PSP/GPDH bidomain enzymes of 10 chlorophytes. Bootstrap values less than 65% are not shown.

To better understand the evolution of the *Chlamydomonas* bidomain proteins, a phylogenetic tree of the conserved PSP/GPDH domains was constructed by the neighbor-joining method (**Figure 3**). The tree was rooted on homologous proteins from two non-chlorophytes, a rhodophyte (*Porphyridium purpureum*) and a primitive relative of the fungi (*Sphaeroforma arctica*) that were recently submitted to GenBank. Within each *Chlamydomonas* species, the isoforms clustered with high bootstrap values, suggesting that within each species, the isoforms diverged from a single gene. *C. reinhardtii*, Chlamy-UWO and Chlamy-ICE formed a group that clusters separately from *Dunaliella*, but otherwise the three *Chlamydomonas* clusters appear weakly related, in agreement with the considerable differences in exon structures of the three species. These findings, together with the finding of the *P. purpureum* protein, raise the possibility that the chlorophyte bidomain proteins have an ancient origin, possibly dating back to a common ancestor of the chlorophytes and rhodophytes.

## Discussion

In view of the importance of glycerol as a compatible solute in animal and plant physiology, it is surprising that so little was known about how it was synthesized until recently. Previous studies referred to the enzymes involved only generically without identifying specific genes, and thus not really “nailing down” the pathway. Recently, the specific enzymes involved in glycerol synthesis have been identified in a bacterium (Larrouy-Maumus et al., 2013), a fish (Raymond, 2015), and, as highlighted here, two species of algae (Morales-Sanchez et al., 2017; He Q. H. et al., 2020). The algal enzymes are especially interesting because of their novel form in which the two enzymes needed to convert DHAP into glycerol are fused into a single enzyme. Although these double enzymes are largely limited to chlorophytes, the recent finding of homologous genes in two unicellular organisms (*P. purpureum* and *S. arctica*) that have links to some of the earliest eukaryotes (Mendoza et al., 2002; Bhattacharya et al., 2013) suggests that the fused gene may have evolved early in the history of eukaryotes.

The present results show that multiple copies of similar enzymes are found in two glycerol-producing *Chlamydomonas* species from Antarctica. Furthermore, the expression of a representative isoform from Chlamy-UWO was strongly upregulated by high salinity and the upregulation was associated with an increase in glycerol production. This result was supported by a recent proteomic study of Chlamy-UWO in which enzymes classified as NAD^+^-dependent GPDHs showed a six-fold increase when NaCl concentration was increased from near zero to 700 mM (Kalra et al., 2020). These results indicate that the bidomain enzyme is a major source of glycerol in Chlamy-UWO, and most likely in Chlamy-ICE as well. Using glycerol as an osmoprotectant, Chlamy-UWO and Chlamy-ICE can survive in salinities greater than the salinity of seawater (at least 700 and 1300 mM NaCl for Chlamy-ICE and Chlamy-UWO, respectively; equivalent to about 1.4x and 2.5x the salinity of seawater, respectively). The highest glycerol levels observed in Chlamy-ICE and Chlamy-UWO were 150 and 420 mM, respectively (**Figure 1A**). (Chlamy-ICE may not need higher levels of glycerol since it appears to live in shallower and thus less saline waters than Chlamy-UWO). For comparison, *C. reinhardtii*, a non-halotolerant species, can tolerate a maximum salinity of 200 mM NaCl (León and Galván, 1994) (but no more than 100 mM NaCl in our laboratory) and accumulates only about 26 mM glycerol in medium containing 100 mM KCl. (Husic and Tolbert, 1986). On the other hand, the marine coastal *C. pulsatilla* can produce much higher levels of glycerol, 1,450 mM when grown in double-strength seawater (Ahmad and Hellebust, 1986).

It should be noted that the glycerol levels in the Lake Bonney species are not enough to balance the osmolarity of the external medium. For example, at 1,300 mM NaCl (~2.6 Osm), the glycerol level in Chlamy-UWO was only about 0.4 Osm. Thus, the cells appear to be increasing the concentrations of additional osmolytes to maintain osmotic equilibrium. In addition to glycerol, proline and sucrose were found to accumulate at high levels in high-salt-grown Chlamy-UWO cultures (Kalra et al., 2020). In *C. pulsatilla*, in which glycerol contributed only about 57% to the intracellular osmolality, large increases in sodium and chloride ions made important contributions to maintaining osmotic equilibrium, but sugars and amino acids did not appear to have a significant role (Ahmad and Hellebust, 1986).

*C. reinhardtii* has four other GPDHs designated GPD1, GPD4, GPD5, and the mitochondrial mtGPD (Morales-Sanchez et al., 2017). tBlastn analyses of contigs assembled from the Chlamy-UWO and Chlamy-ICE transcriptomes indicate that both species have close homologs of each of these proteins (data not shown). We cannot rule out the possibility that these GPDHs also have a role in glycerol production, although GPD4 in *C. reinhardtii* was not upregulated by 125 mM NaCl (Morales-Sanchez et al., 2017).

That all the Lake Bonney PSP/GPDH isoforms have a chloroplast targeting signal suggests that they function in the chloroplast, as was concluded for the bidomain enzymes in *C. reinhardtii* (Morales-Sanchez et al., 2017) and *D. salina* (He Q. H. et al., 2020). This seems reasonable in view of the fact that the enzymes could act directly on DHAP produced by the Calvin cycle in the chloroplast. However, several chlorophyte bidomain proteins in the databank do not appear to have chloroplast signals as judged by ChloroP, which suggests they function in the cytoplasm.

Because Chlamy-UWO and Chlamy-ICE are living in an environment that is constantly exposed to freezing, as demonstrated by their expression of numerous ice-binding proteins (Raymond and Morgan-Kiss, 2013; Raymond and Morgan-Kiss, 2017), glycerol, by lowering the freezing point of the intracellular medium, could also act as a cryoprotectant. These might be the only organisms to use glycerol as both an osmoprotectant and a cryoprotectant.

Most of the known bidomain glycerol enzymes belong to chlorophytes, which in addition to *Chlamydomonas* and *Dunaliella*, include *Gonium*, *Raphidocelis*, *Micractinium*, *Chlorella*, *Chloropico*n, *Volvox*, and *Tetrabaena*. Many of these species are freshwater species and so it remains to be seen whether these species also produce glycerol and for what purpose. It also remains to be seen whether other marine *Chlamydomonas* species that make glycerol (Ahmad and Hellebust, 1986; Miyasaka et al., 1998) also have these enzymes. Finally, little is known about how *Chlamydomonas* spp. sense changes in salinity. Recent studies on osmosensing in microalgae (Suescún-Bolívar and Thomé, 2015; Charneco et al., 2018; He Q. et al., 2020) have implicated the possible involvement of mitogen-activated protein (MAP) kinases. The behavior of these genes in response to increases in salinity should thus be interesting.

In summary, we show that two Antarctic extremophiles, in addition to having several adaptations to low light and low temperature, also have adapted to high salinity by producing glycerol as an osmoprotectant (and possibly a cryoprotectant), and at least one of them (Chlamy-UWO) achieves this by using an unusual bidomain enzyme that can make glycerol directly from DHAP.

## Data Availability Statement

Publicly available datasets were analyzed in this study. The Chlamydomonas sp. UWO241 transcriptome is available under GenBank accession PRJNA575885.

## Author Contributions

All authors contributed equally to this study.

## Funding

This study was partially funded by NSF grant 1637708 and DOE Grant DE-SC0019138 to RM-K.

## Conflict of Interest

The authors declare that the research was conducted in the absence of any commercial or financial relationships that could be construed as a potential conflict of interest.

## Supplementary Material

Table S1 qPCR primers used in this study.

Figure S1 Alignment of PSP/GPDH domains of Chlamy-ICE and *Dunaliella salina* showing conservation of ligand binding sites. The binding sites in *Dunaliella* are from He Q. H. et al. (2020). Red underline, PSP domain; blue underline, GPDH domain. Blue asterisks, glycerol-3 phosphate; green asterisks, NAD; red asterisks, DHAP. Black background, identical a.a.s; gray background, similar a.a.s. Numbering refers to position in the Chlamy-ICE protein. The region shown (from T60 to F639) is the same region shown in **Figure 2B**.

Figure S2 Stereo views comparing the ligand binding sites in the GPDH domain of the PSP/GPDH protein in *Dunaliella salina* (top) and Chlamy-UWO isoform 2 (bottom). The *Dunaliella* structure was determined by X-ray crystallography. The Chlamy-UWO structure is predicted by SwissModel based on the *Dunaliella* structure. The binding sites for *Dunaliella* were obtained from He Q. H. et al. (2020). The amino acid residues forming the binding sites are shown. Red, DHAP binding site; green, NAD binding site. These amino acid residues correspond to the red and green asterisks, respectively, in **Figure S1**.
