# Supplementary material for: Glycerol Is an Osmoprotectant in Two Antarctic Chlamydomonas Species From an Ice-Covered Saline Lake and Is Synthesized by an Unusual Bidomain Enzyme
Source: Front Plant Sci. 2020 Aug 20;11:1259. doi: 10.3389/fpls.2020.01259 (PMC7468427; doi:10.3389/fpls.2020.01259)
Supplement: Table S1 — qPCR primers used in this study. [file Table_1.docx]

Table S1. qPCR primers used in this study

| Gene | Protein Name | Primer Sequence | Size (bp) |
| --- | --- | --- | --- |
| *rps10* | Ribosomal | F 5’ ACCACTCTTTCACCGTCA 3’ | 172 |
|  | protein S10 | R 5’ TTTGTAGATTGCCCTCCTG 3’ |  |
| *histh2b* | Histone H2B | F 5’CCTTCATCAACGACATCT 3’  R 5’GGGAGAATGAGGCGGATT 3’ | 117 |
| *PSP* | Phosphoserine phosphatase/GDPH | F 5’AGCGCCAAAGACAACCAA 3’  R 5’ CATGGCCTGGTTTGTGAT 3’ | 212 |
