# Supplementary material for: Glycerol Is an Osmoprotectant in Two Antarctic Chlamydomonas Species From an Ice-Covered Saline Lake and Is Synthesized by an Unusual Bidomain Enzyme
Source: Front Plant Sci. 2020 Aug 20;11:1259. doi: 10.3389/fpls.2020.01259 (PMC7468427; doi:10.3389/fpls.2020.01259)
Supplement: Figure S1 — Alignment of PSP/GPDH domains of Chlamy-ICE and Dunaliella salina showing conservation of ligand binding sites. The binding sites in Dunaliella are from He Q. H. et al. (2020). Red underline, PSP domain; blue underline, GPDH domain. Blue asterisks, glycerol-3 phosphate; green asterisks, NAD; red asterisks, DHAP. Black background, identical a.a.s; gray background, similar a.a.s. Numbering refers to position in the Chlamy-ICE protein. The region shown (from T60 to F639) is the same region shown in Figure 2B. [file Presentation_1.zip › supplementary figure 2.DOCX]

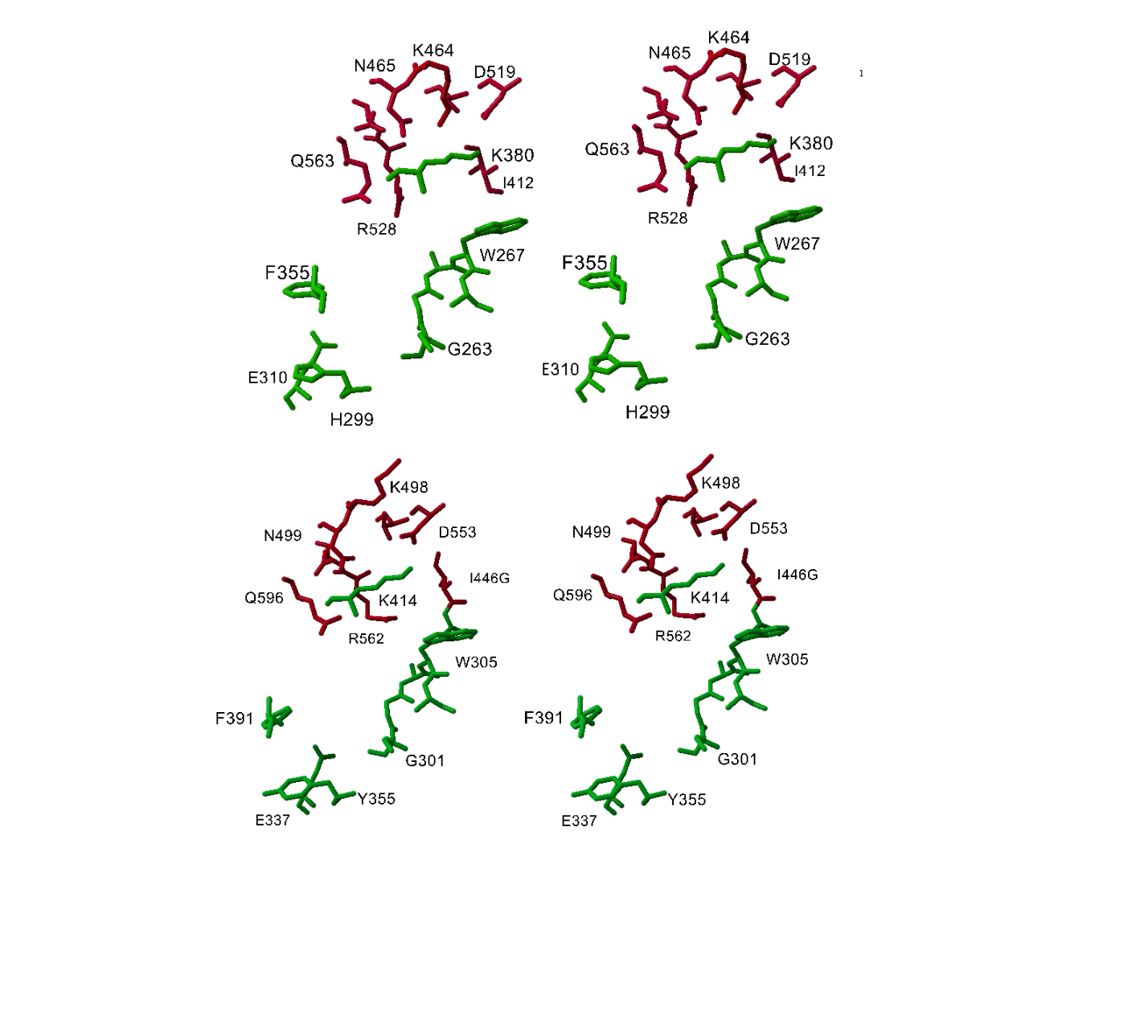


Fig. S2. Stereo views comparing the ligand binding sites in the GPDH domain of the PSP/GPDH protein in *Dunaliella salina* (top) and Chlamy-ICE isoform 2 (bottom). The *Dunaliella* structure was determined by X-ray crystallography. The Chlamy-ICE structure is predicted by SwissModel based on the *Dunaliella* structure. The binding sites for *Dunaliella* were obtained from He et al. (2019). The amino acid residues forming the binding sites are shown. Red, DHAP binding site; green, NAD binding site. These amino acid residues correspond to the red and green asterisks, respectively, in Fig. S1.
